# Advances and Barriers in Understanding Presynaptic *N*-Methyl-*D*-Aspartate Receptors in Spinal Pain Processing

**DOI:** 10.3389/fnmol.2022.864502

**Published:** 2022-03-31

**Authors:** Annemarie Dedek, Michael E. Hildebrand

**Affiliations:** ^1^Department of Neuroscience, Carleton University, Ottawa, ON, Canada; ^2^Neuroscience Department, Ottawa Hospital Research Institute, Ottawa, ON, Canada

**Keywords:** dorsal horn, primary afferent, presynaptic, NMDAR, pain, spinal cord, sex differences, developmental timepoints

## Abstract

For decades, *N*-methyl-*D*-aspartate (NMDA) receptors have been known to play a critical role in the modulation of both acute and chronic pain. Of particular interest are NMDA receptors expressed in the superficial dorsal horn (SDH) of the spinal cord, which houses the nociceptive processing circuits of the spinal cord. In the SDH, NMDA receptors undergo potentiation and increases in the trafficking of receptors to the synapse, both of which contribute to increases in excitability and plastic increases in nociceptive output from the SDH to the brain. Research efforts have primarily focused on postsynaptic NMDA receptors, despite findings that presynaptic NMDA receptors can undergo similar plastic changes to their postsynaptic counterparts. Recent technological advances have been pivotal in the discovery of mechanisms of plastic changes in presynaptic NMDA receptors within the SDH. Here, we highlight these recent advances in the understanding of presynaptic NMDA receptor physiology and their modulation in models of chronic pain. We discuss the role of specific NMDA receptor subunits in presynaptic membranes of nociceptive afferents and local SDH interneurons, including their modulation across pain modalities. Furthermore, we discuss how barriers such as lack of sex-inclusive research and differences in neurodevelopmental timepoints have complicated investigations into the roles of NMDA receptors in pathological pain states. A more complete understanding of presynaptic NMDA receptor function and modulation across pain states is needed to shed light on potential new therapeutic treatments for chronic pain.

## Introduction

### Pain

Acute pain is a critical protective mechanism that alerts the body to tissue damage. The somatosensory nociceptive system is comprised of peripheral sensory neurons, circuits in the superficial dorsal horn (SDH) of the spinal cord and many target brain regions. The peripheral sensory neurons and the SDH are responsible for transducing and modulating nociceptive input; once afferent signals are processed in the brain, conscious perception of nociception results in the multifaceted experience of pain. As a complex network of brain areas are involved in integrating and modulating pain [for review see Almeida et al. (2004)], a practical treatment approach is to target nociceptive input before it reaches the brain.

### Nociception in the Spinal Dorsal Horn

The cellular organization of the SDH lends itself to plastic changes that can increase network excitability and nociceptive output. Lamina I and II, the outer-most laminae of the SDH, are the main sites of entry for high-threshold nociceptive primary afferents (Todd, 2010; Peirs and Seal, 2016; Peirs et al., 2021). Aδ fibers are small-diameter, myelinated primary afferent fibers that synapse onto lamina I and lamina II_outer_ neurons. C fibers also have a small diameter but are unmyelinated and synapse onto lamina I-II neurons. Most neurons in laminae I and III, and virtually all neurons in lamina II, are interneurons, meaning that they make local synaptic connections exclusively within the SDH. Interneurons can be divided into two general subtypes: excitatory (glutamatergic) and inhibitory [GABAergic (γ-aminobutyric acid-ergic) and/or glycinergic] (Todd, 2017). In addition to input from primary afferents, the SDH receives input from descending efferents from the brain (Kato et al., 2006; Todd, 2010, 2015). The final neuronal subpopulation within the SDH is a small number of projection neurons (Todd, 2015). Given this convergence of interconnected synaptic inputs from primary afferents, descending efferents, and local interneurons onto SDH projection neurons, targeting molecular determinants of excitability within this network can readily lead to altered nociceptive output.

### *N*-Methyl-*D*-Aspartate Receptors in Spinal Nociception and Hyperexcitability

Excitatory glutamatergic *N*-methyl-*D*-aspartate receptors (NMDARs) are critical regulators of SDH plasticity and excitability. The seminal discovery of windup, which is characterized by progressively increased amplitude of SDH neuronal depolarization and firing during a course of repeated C-fiber stimulation (Mendell and Wall, 1965), paved the way for investigating the role of NMDARs in acute nociceptive processing. Both competitive and non-competitive NMDAR antagonists can block windup (Woolf and Thompson, 1991). Selective NMDAR antagonists also reverse increased excitability in SDH neurons in the subcutaneous formalin injection model of acute pain (Coderre and Melzack, 1992).

Dysregulated processing of nociceptive input results in pathological pain through hyperexcitability of the SDH (Woolf, 2011), which is dependent upon NMDAR activation (Bourinet et al., 2014). Early studies found that intrathecal injection of the NMDAR antagonist MK-801 reduced mechanical and heat hyperalgesia, while leaving acute nociception unaltered in sham-treated animals (Chapman and Dickenson, 1992; Yamamoto and Yaksh, 1992a,b). In later studies, selective knockdown of the obligatory NMDAR subunit, GluN1, in the SDH through intrathecal viral injections prevented the induction of pain hypersensitivity induced by injury but did not affect pain thresholds in uninjured animals (South et al., 2003; Garraway et al., 2007). Importantly, NMDARs can be located on both the pre- and postsynaptic membrane, and yet early research into the nociceptive roles of NMDARs has not made this critical distinction between pre- and postsynaptic receptors in the SDH.

### Purpose

Historically, the role of presynaptic NMDARs has been overshadowed by their postsynaptic counterparts. For example, many of the processes mediating chronic pain have exclusively focused on postsynaptic changes. In recent years, it has become evident that presynaptic NMDARs (preNMDARs) contribute to the etiology of pathological pain. Here, we will explore how preNMDARs are modulated in the SDH and contribute to pain signaling. Further, we will highlight gaps in the literature regarding the nociceptive roles of preNMDARs.

## Presynaptic *N*-Methyl-*D*-Aspartate Receptors

Studies in the 1990s discovered that exogenous application of NMDA resulted in increased neurotransmitter release from monoaminergic terminals in the striatum (Krebs et al., 1991). This finding provided some of the first evidence for functional preNMDARs that modulate the release of neurotransmitters (Krebs et al., 1991). Electron microscopy with an antibody targeting the GluN1 NMDAR subunit in male adult Sprague Dawley rats showed extensive labeling of the presynaptic terminal in the SDH, revealing for the first time that NMDARs are present presynaptically in the dorsal horn of the spinal cord (Liu et al., 1994). In the SDH, nearly one-third of NMDARs are found to be presynaptic and are located immediately adjacent to the vesicle release site at the active zone (Liu et al., 1994). PreNMDARs in primary afferent terminals are translated in dorsal root ganglia neurons and are transported along the axon to the afferent terminal (Liu et al., 1994). The localization of NMDARs in the axon terminals of primary afferents, adjacent to the vesicle release site, allows them to influence the release of glutamate and peptide neurotransmitters from primary afferents, thus directly modulating the first nociceptive input to the central nervous system (Liu et al., 1994; Duguid and Smart, 2008; Abraira et al., 2017; Zimmerman et al., 2019).

### Properties of Presynaptic *N*-Methyl-*D*-Aspartate Receptors

The type of NMDAR subunits that make up receptors in the presynaptic terminal gives insight into the function of these receptors, as well as an opportunity to selectively inhibit a subpopulation of NMDARs. Interestingly, all subunit types (GluN2A-D and GluN3A-B) can assemble into preNMDARs in the nervous system (Bouvier et al., 2015; Oshima-Takago and Takago, 2017). Primary afferent terminals have been found to contain functional GluN2B subunits in adult rats (Boyce et al., 1999; Chen et al., 2010; Zhao et al., 2012; Yan et al., 2013). However, further studies are needed to fully characterize the expression of specific subunits in preNMDARs in the spinal cord.

**FIGURE 1:**
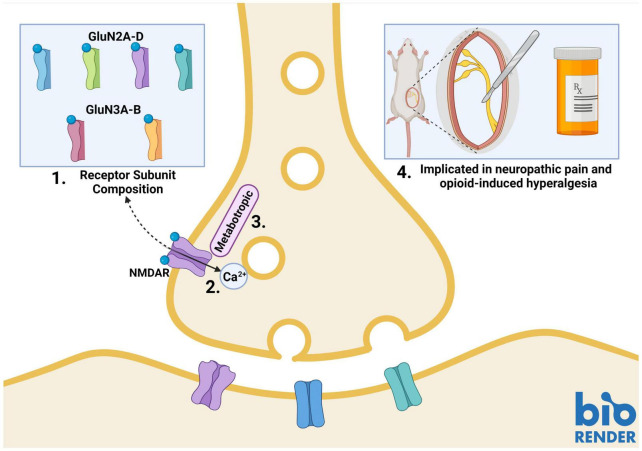
Properties of presynaptic *N*-methyl-*D*-aspartate receptors (preNMDARs) in the superficial dorsal horn (SDH). 1. GluN2A and GluN2B-containing preNMDARs in primary afferents have been implicated in pain models. Low Mg^2+^ sensitivity suggests a potential role of GluN2D or GluN3-containing preNMDARs. 2. PreNMDARs may contribute to signaling in the SDH by either direct cation influx, or 3. By metabotropic signaling. 4. To date, preNMDARs have been found to contribute to both neuropathic pain and opioid-induced hyperalgesia, but not to non-pathological nociception. Figure was created using BioRender.com.

Presynaptic NMDARs have different properties than their postsynaptic counterparts. Canonical postsynaptic NMDARs are blocked by Mg^2+^ at rest; they, therefore, require neuronal depolarization along with glutamate binding to become functionally active. Presynaptic NMDARs, on the other hand, can promote spontaneous neurotransmitter release in the absence of neuronal depolarization (Kavalali, 2015; Dore et al., 2017; Wong et al., 2021). This suggests that these NMDARs can be activated by endogenous glutamate without relief of the Mg^2+^ block (Mayer et al., 1984; Kunz et al., 2013; Kavalali, 2015). Studies in the cortex and hippocampus have identified GluN2C/GluN2D subunit-containing preNMDARs, which convey low Mg^2+^ sensitivity and low calcium-permeability to the NMDAR (Banerjee et al., 2009; Andrade-Talavera et al., 2016). Studies of NMDAR subunit expression patterns have identified GluN2D expression in the spinal cord (Dunah et al., 1996; Temi et al., 2021), however, further research is needed to identify if these subunits are incorporated into SDH preNMDARs. PreNMDARs in the visual cortex have been found to contain GluN1/GluN3A/GluN2B triheteromers (Larsen et al., 2011). This type of triheteromer is presumed to be less sensitive to Mg^2+^ and have lower Ca^2+^ permeability than diheteromeric GluN1/GluN2B NMDARs (Paoletti et al., 2013). GluN3A subunits have also been found in the spinal cord, however, it is unknown if they are present in SDH synaptic terminals (Figure 1; Kehoe et al., 2013).

Another factor that may explain preNMDAR Mg^2+^ insensitivity and associated functions in the absence of neuronal depolarization is metabotropic NMDAR signaling (Dore et al., 2017; Wong et al., 2021). In addition to ionotropic functions, NMDARs can signal via ion-flux-independent mechanisms (Dore et al., 2015, 2017). Metabotropic preNMDAR signaling has recently come to light during investigations into differences in mechanisms governing spontaneous and evoked vesicle release. Contrary to classical theories of synaptic transmission, the release of vesicles in spontaneous and evoked neurotransmission may occur by independent mechanisms that rely on different types of NMDAR function. Abrahamsson and colleagues found that, in pyramidal cells of the visual cortex, Mg^2+^-sensitive preNMDARs upregulate the readily-releasable vesicle pool during high-frequency firing through Rab3-interacting molecules (RIMs), which provide scaffolding at presynaptic active zones. Conditional RIM1αβ deletion abolished the upregulation of vesicle replenishment, but preNMDAR-mediated spontaneous vesicle release was unaffected, even when preNMDAR ionotropic function was blocked by the channel-pore blocker MK-801 (Abrahamsson et al., 2017). Spontaneous vesicle release was found to be mediated by Mg^2+^-insensitive preNMDARs that signal metabotropically through c-Jun *N*-terminal kinase (JNK), indicating that evoked and spontaneous vesicle release occurs through distinct processes (Figure 1; Abrahamsson et al., 2017; Bouvier et al., 2018).

## Presynaptic Modulation of Plasticity by Presynaptic *N*-Methyl-*D*-Aspartate Receptors

The trafficking and function of NMDARs are regulated by protein tyrosine kinases such as Src-family kinases (SFKs). Indeed, SFK-dependent phosphorylation of NMDARs has been intricately tied to SDH plasticity (Xie et al., 2016; Suo et al., 2017) as well as the development of both inflammatory and neuropathic pain (Salter and Kalia, 2004; Liu et al., 2008; Sorge et al., 2011; Hildebrand et al., 2014, 2016). Although most studies have focused on NMDAR modulation exclusively at postsynaptic sites, SFKs have also been implicated in regulating preNMDARs in primary afferents. PreNMDAR activity at primary afferent terminals can be indirectly measured by neurokinin 1 (NK1) receptor internalization, which occurs as a result of presynaptic release of substance P (SP) (Marviźon et al., 1997). High-frequency stimulation (100 Hz) of the dorsal root induces the release of SP (a nociception-specific neuropeptide) and subsequent NK1 receptor internalization in NK1R-positive neurons in laminae I and II-outer. This NK1R internalization is also induced by administering NMDA and is abolished using AP5, a selective NMDAR antagonist, but is unaffected by the AMPAR and kainate receptor antagonist, CNQX, indicating that SP release in the SDH is regulated by NMDARs (Marviźon et al., 1997). Interestingly, NMDA-induced SP release does not require the firing of primary afferents or the opening of Ca^2+^ channels, supporting the notion that preNMDARs result in SP release via their own influx of Ca^2+^ into primary afferent terminals (Chen et al., 2010) or through metabotropic signaling (Dore et al., 2015; Bouvier et al., 2018). PreNMDAR-mediated SP release can be attenuated by blocking SFKs using PP1 or dasatinib (Chen et al., 2010). In addition, another study examining the role of SFKs in SP release found that intrathecal administration of NMDA only caused NK1 internalization when pretreated with BDNF (Chen W. et al., 2014). To investigate the role of SFKs in NK1 internalization, Chen W. et al. (2014) incubated spinal cord slices in BDNF for 60 minutes, followed by incubation in NMDA and either dasatinib, PP2 (both are SFK inhibitors), or PP3 (an inactive PP2 analog). They found that NK1R internalization resulting from preNMDAR-mediated SP release is blocked by inhibiting SFKs (using PP2 or dasatinib) and is unaffected by PP3 (Chen W. et al., 2014). Moreover, in mossy fibers of dentate granule cells, preNMDAR activation results in BDNF release from axon terminals, further promoting plastic changes (Lituma et al., 2021). These results suggest that regulating SFKs in primary afferent terminals could help modulate aberrant incoming nociceptive signals to the CNS in pathological pain states.

## Role of Presynaptic *N*-Methyl-*D*-Aspartate Receptors in Pathological Pain Models

### Presynaptic *N*-Methyl-*D*-Aspartate Receptors in Neuropathic Pain Models

Neuropathic pain is chronic pain that occurs following damage to neurons in the nociceptive pathway. To understand the role of preNMDARs in neuropathic pain, Yan et al. (2013) examined adolescent male rats subjected to the spinal nerve ligation (SNL) model of neuropathic pain. They found that evoked EPSC amplitudes in neuropathic rats were higher than sham animals. They also found a decrease in the paired-pulse ratio of neuropathic rats, indicating that SNL increased the probability of neurotransmitter release from presynaptic terminals (Yan et al., 2013). PreNMDARs that are upregulated in this model were found to be predominantly GluN2B subunit-containing (Figure 1; Yan et al., 2013). This has interesting implications, as studies have found that GluN2B NMDARs mediate baseline dorsal horn postsynaptic NMDAR responses in naïve rats as well as spinal hyperexcitability in a neuropathic pain model, but these studies did not examine the role of GluN2B-containing preNMDARs (Hildebrand et al., 2014, 2016; Tong and MacDermott, 2014). Increases in preNMDAR activation are dependent on activation of protein kinase C (PKC), as demonstrated by reversal with the PKC inhibitor GF109203 in spinal slices from SNL rats (Yan et al., 2013). Activation of preNMDARs was also linked to increased release of substance P, increased frequency of miniature EPSCs and associated pain hypersensitivity in multiple models of neuropathic pain, including the chronic constriction injury (CCI) and SNL models, in adolescent or young adult male rats (Chen W. et al., 2014; Chen S. R. et al., 2014; Li et al., 2016).

In addition to the CCI and SNL models of neuropathic pain, preNMDARs have been implicated in the development of paclitaxel-induced neuropathic pain in male rats. Xie and colleagues found similar preNMDAR-induced increases in mEPSC frequency and a reduction in paired-pulse ratio in paclitaxel-treated rats (Xie et al., 2016). However, unlike the peripheral nerve injury models (Yan et al., 2013), the increase in preNMDAR activity by PKC in the paclitaxel model was mediated through phosphorylation of GluN2A-containing NMDARs (Xie et al., 2016) instead of GluN2B. Interestingly, glutamate release in sham animals was not regulated by presynaptic NMDARs in any of the above-described experiments. This suggests that neuropathic injury results in recruitment of preNMDAR regulation pathways to enhance glutamate release and drive an increase in excitability in the SDH and that preNMDARs are not involved in neurotransmitter release in uninjured male adolescent animals (Figure 1; Yan et al., 2013; Li et al., 2016; Xie et al., 2016). In support of this, selective knockdown of primary afferent NMDARs did not affect phase I, the acute phase, of the formalin model of pain (McRoberts et al., 2011). Importantly, presynaptic glutamate receptors may still be a target of anesthetics (Kubota et al., 2020), but further research is needed to understand the role of preNMDARs in non-pathological nociceptive SDH signaling.

Gabapentin is a common pain therapeutic; although it has been used for decades to treat pain, new mechanisms of action are still being uncovered (Kukkar et al., 2013). One of gabapentin’s targets, α2δ-1, a voltage-activated calcium channel subunit, has recently been shown to interact with preNMDARs to augment glutamatergic input to the SDH. Nerve injury increases the expression of α2δ-1 in the DRG and spinal dorsal horn (Luo et al., 2001; Huang et al., 2019). Recent studies have shown that the C-terminal of α2δ-1 interacts with preNMDARs to promote synaptic/plasma membrane trafficking of α2δ-1-bound NMDARs (Chen et al., 2018; Huang et al., 2020). Blocking NMDARs reverses the SNL-mediated over-expression of α2δ-1 and the increase in frequency of mEPSCs in male rats. Gabapentin disrupts the interaction of α2δ-1 with NMDARs and thus blocks nerve injury-induced potentiation of presynaptic and postsynaptic NMDAR activity in the SDH (Chen et al., 2018; Zhang et al., 2021). Interestingly, this suggests clinical use of gabapentin may be modulating preNMDARs in human neuropathic pain.

### Presynaptic *N*-Methyl-*D*-Aspartate Receptors in Opioid-Induced Hyperalgesia

Prolonged exposure to opioids results in paradoxical opioid-induced hyperalgesia. This phenomenon has long been tied to NMDAR signaling. In a study on the role of preNMDARs in opioid-induced hyperalgesia, Zeng et al. performed whole-cell patch clamp recordings on lamina I neurons while using the NMDAR antagonist MK-801 to block postsynaptic NMDARs. They perfused NMDA onto spinal slices from unsexed opioid-tolerant juvenile rats and found an increase in mEPSC frequency. Morphine-tolerant animals also had increased numbers of SDH primary afferents containing NMDARs, as determined using immunogold labeling of GluN1 subunits and electron microscopy (Zeng et al., 2006). Furthermore, the effects of preNMDARs on opioid tolerance was blocked by PKC inhibitors in male rats, suggesting that PKC may be potentiating preNMDARs to increase glutamate, resulting in opioid-induced hypersensitivity (Zhao et al., 2012). Moreover, co-administration of NMDAR antagonists with opioids also attenuates the development of opioid tolerance (Figure 1; Chapman and Dickenson, 1992; Zeng et al., 2006; Shen et al., 2018).

### Inflammatory Pain

Presynaptic *N*-methyl-*D*-aspartate receptors are known to play a role in both neuropathic pain and opioid-induced hyperalgesia, however, they do not seem to be involved in inflammatory pain. In a study examining the effects of inflammatory pain modeled by plantar injection of complete Freud’s adjuvant (CFA) in young adult male rats, increased glutamatergic input to lamina I (but not lamina II) was found to occur via presynaptic TRPA1 and TRPV1, as opposed to preNMDARs (Deng et al., 2019; Huang et al., 2019). This study looked at animals 10-16 days following injection of CFA. This is noteworthy because Weyerbacher and colleagues have previously shown, using both sexes of mice, that the maintenance of inflammatory pain occurs via an NMDAR-independent mechanism. They show, using knockout of GluN1 subunits in SDH neurons, that 96 h post-CFA injection, knockout animals are no longer protected from the effects of CFA, indicating that although NMDARs are involved in the development of inflammatory pain, they are not involved in its maintenance (Weyerbacher et al., 2010). Unfortunately, this study did not specifically evaluate the role of preNMDARs. To investigate the potential role of preNMDARs in the initiation of inflammatory pain hypersensitivity, future studies that include timepoints during the “induction phase” (1–3 days post-CFA injection) (Fehrenbacher et al., 2012); of CFA-induced inflammatory pain are needed.

## Unanswered Questions on Presynaptic *N*-Methyl-*D*-Aspartate Receptors

### Presynaptic *N*-Methyl-*D*-Aspartate Receptors Across Development

The expression pattern of NMDARs in the CNS varies across the lifespan, enabling distinct NMDAR subunit-specific mechanisms of plasticity at discrete developmental stages. For example, in the prenatal and early postnatal brain, there is a high expression of GluN2B and GluN2D NMDARs (Crair and Malenka, 1995; Hsia et al., 1998). The properties of GluN2B and GluN2D subunits promote temporal summation and integration at developing synapses due to their slow deactivation kinetics (Paoletti et al., 2013). In the weeks following birth, there is a developmental switch in the brain that promotes GluN2A-NMDAR expression, while expression and synaptic localization of GluN2B and GluN2D are decreased. This results in decreased synaptic strength and dampens the probability of further NMDAR-dependent functional circuit reorganization in the adult brain (Gray et al., 2011). However, the GluN2B/GluN2D to GluN2A developmental switch does not occur in the SDH of male rats (females have not been studied) (Mahmoud et al., 2020). Instead, the relative contributions of GluN2A- and GluN2B-mediated NMDAR postsynaptic responses at lamina II synapses remain constant throughout early postnatal development in male rats (Mahmoud et al., 2020). Consistent with this, the GluN2A, GluN2B, and GluN2D subunits are all found to be expressed in the SDH of male and female juvenile rats (Temi et al., 2021). However, many foundational findings from both the brain and spinal cord do not separate pre- from postsynaptic NMDARs, which is problematic given the differential functions of these populations in synaptic physiology.

**FIGURE 2:**
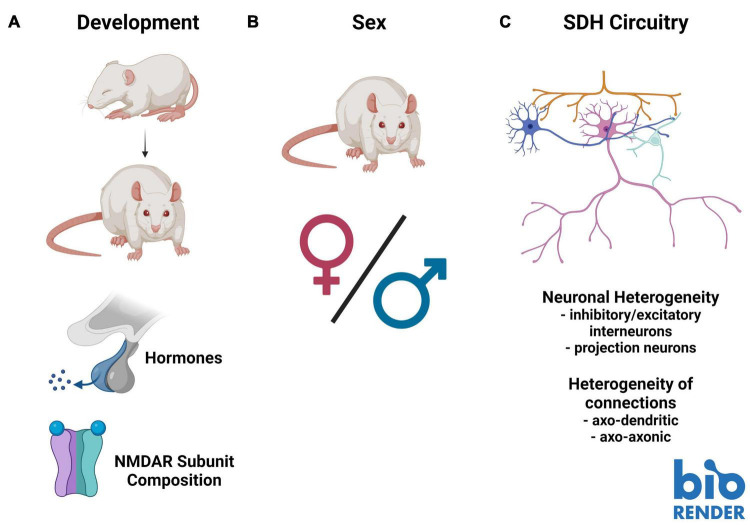
Barriers to understanding the role of presynaptic *N*-methyl-*D*-aspartate receptors (preNMDARs) in superficial dorsal horn (SDH) nociception. **(A)**. Existing literature on preNMDARs in the SDH spans several developmental timepoints. This makes comparison between studies problematic, as NMDAR expression and composition may change throughout development. **(B)**. Almost all studies on preNMDARs in the SDH have been conducted in male or unsexed animals. Because data in recent years has demonstrated substantial sex differences in not only pain and nociception, but in the role of NMDARs in pain, it is critical for future studies to examine preNMDARs in females. **(C)**. The complex circuitry and unknown connectivity of the SDH makes interpreting overall output from the contributions of preNMDARs difficult. Increased mapping of the connectivity of distinct neuronal subpopulations, such as primary afferent terminals, inhibitory and excitatory interneurons, as well as projection neurons, in addition to understanding the distinct contributions of axo-dendritic and axo-axonic connections, will be vital to fully understand the role of preNMDARs in nociception in the SDH. Figure was created using BioRender.com.

Of the available evidence on preNMDARs in nociceptive processing, results from early postnatal animals appear to contrast with findings from adolescent and adult models. For example, inhibiting NMDARs in the SDH of SNL or opioid-tolerant adult rats reduced the frequency of mEPSCs, and did not affect mEPSC frequency in naïve animals (Zhao et al., 2012; Yan et al., 2013). This suggests that in mature animals preNMDARs increase glutamatergic vesicle release in some pathological pain states, and do not affect baseline transmission. However, in naïve postnatal day 12–17 rats, Zeng and colleagues found that application of NMDA to activate preNMDARs reduced mEPSC frequency and reduced the amplitude of evoked EPSCs (Zeng et al., 2006). Additionally, Bardoni et al. (2004) showed that bath application of NMDA to spinal slices from postnatal day 6–12 rats caused an increase in synaptic latency, as well as the failure of monosynaptic AMPAR EPSCs in lamina I, with some heterogeneity in responses between individual neurons (Bardoni et al., 2004). They also found that NMDA application decreased the amplitude of the AMPAR EPSCs, which was attenuated by the NMDAR antagonist D-APV, suggesting that preNMDAR stimulation decreases glutamate release in these young animals (Bardoni et al., 2004). Since NMDAR expression and subunit composition varies greatly throughout development (Ewald and Cline, 2008), studies examining preNMDAR function across development can address the disparity between these studies. This is necessary for a complete understanding of the role preNMDARs play in the control of neurotransmitter release both in normal physiological conditions and in pathological pain (Figure 2). An important clinical consideration relating to these studies is that specific pain conditions affect patients of discrete ages. For example, osteoarthritis primarily affects older adults (Zhang and Jordan, 2010), and thus, adult rodent models best represent that pathology (Bapat et al., 2018). The varied function of NMDARs across development, combined with age-specific pain pathologies requires using age-appropriate rodent models to accurately study preNMDAR contribution to pathological pain.

### Potential Sex Differences in Pain-Processing Presynaptic *N*-Methyl-*D*-Aspartate Receptors

Building on age-related developmental differences in NMDAR function, recent studies have highlighted the importance of considering sex and sex-hormone-related factors in pain models (Sorge et al., 2015; Mapplebeck et al., 2019; Dedek et al., 2021). Specifically, some of these differences have been tied to NMDAR function and regulation (Mogil et al., 1993; Dedek et al., 2021). For example, we found a sex-hormone-dependant sexual dimorphism in postsynaptic NMDAR potentiation in CFA-mediated inflammatory and *ex vivo* BDNF-treatment models of pathological pain, which is conserved from rodents to humans (Dedek et al., 2021). Additionally, we identified differences in baseline NMDAR localization within the SDH between male and female juvenile rats: GluN2B and GluN2D are preferentially localized to the SDH in males, but in females only GluN2B is preferentially localized to the SDH, and in males, but not females, GluN2B expression was enhanced in the medial SDH (Temi et al., 2021; Figure 2). Almost the entire body of the above-discussed literature on preNMDARs in the SDH is based on studies in male or unsexed rodents, and thus a critical gap exists in addressing possible sex differences in the roles of SDH preNMDARs in physiological and pathological pain processing.

### Complex Superficial Dorsal Horn Circuitry

A final barrier to fully understanding the role of preNMDARs in mediating nociceptive spinal cord signaling is the complex connectivity making up SDH circuitry. Unlike some other areas of the CNS where circuitry has been mapped out with knowledge of the exact connectivity between defined neuronal populations, like in the hippocampus, the exact connectivity within the SDH is unknown. With high collateralization of primary afferents and a complex network of modulatory interneurons that are either inhibitory or excitatory, inferring the effect on SDH output from specific molecular inputs is extremely difficult (Figure 2). For example, at first glance, it may seem straightforward that an increase in vesicle release from primary afferents would always have an overall excitatory effect in the SDH. However, primary afferents make axo-axonic connections that have been found to result in depression of primary afferent vesicle release. These mechanisms are not yet fully understood (Hochman et al., 2010). In addition, preNMDARs can contribute to the release of a diverse array of neurotransmitter-containing vesicles in either a spontaneous or depolarization-evoked manner (Pittaluga, 2021). Across various regions of the brain, NMDA application results in an increased spontaneous release of dopamine and glutamate, while having no effect on spontaneous GABA release (Pittaluga, 2021). In the SDH, studies performed in juvenile animals suggest a role of preNMDARs in mediating long-term depression (LTD) and presynaptic inhibition of primary afferent terminals (Bardoni et al., 1998, 2004, 2013; Zimmerman et al., 2019; Comitato and Bardoni, 2021), but further investigation is needed across the developmental spectrum. It is also important to consider that the majority of cells in the SDH are locally synapsing excitatory and inhibitory neurons (Todd, 2017), and that preNMDARs are found in the terminals of both of these classes of interneurons (Liu et al., 1994). PreNMDARs are thus likely to regulate the local release of excitatory and inhibitory neurotransmitters within SDH nociceptive networks. For this reason, it is necessary to understand the neuronal identities and connections within the SDH to paint a complete picture of the role of preNMDARs in nociceptive signaling and shed light on potential new therapeutic interventions for the treatment of pain.

## Conclusion

Though preNMDARs have been known to exist for decades, only recently has their contribution to spinal cord physiology come into the spotlight. Thus far, it is clear that preNMDARs play a critical role in modulating the release of glutamate from primary afferents by directly permitting Ca^2+^ entry to the presynaptic terminal and/or by metabotropic signaling. Although progress has been made in understanding the role of preNMDARs in the SDH, several critical barriers remain. Further exploration of potential differences in preNMDARs by sex and across development is needed, as well as integrating this information into the rapidly evolving understanding of the complex molecular and cellular circuitry of the SDH.

## Author Contributions

AD wrote this manuscript, with editing and feedback from MEH. Both authors contributed to the article and approved the submitted version.

## Conflict of Interest

The authors declare that the research was conducted in the absence of any commercial or financial relationships that could be construed as a potential conflict of interest.

## Publisher’s Note

All claims expressed in this article are solely those of the authors and do not necessarily represent those of their affiliated organizations, or those of the publisher, the editors and the reviewers. Any product that may be evaluated in this article, or claim that may be made by its manufacturer, is not guaranteed or endorsed by the publisher.
